# Preparing Wi-Fi 7 for Healthcare Internet-of-Things

**DOI:** 10.3390/s22166209

**Published:** 2022-08-18

**Authors:** Yazdan Ahmad Qadri, Zulqarnain, Ali Nauman, Arslan Musaddiq, Eduard Garcia-Villegas, Sung Won Kim

**Affiliations:** 1 Department of Information and Communication Engineering, Yeungnam University, Gyeongsan-si 38541, Korea; 2 Department of Computer Science and Media Technology, Linnaeus University, 391 82 Kalmar, Sweden; 3 Department of Network Engineering, Universitat Polit’ecnica de Catalunya (UPC), 08034 Barcelona, Spain

**Keywords:** IEEE 802.11be, Internet of Things, orthogonal frequency division multiple access, healthcare

## Abstract

The healthcare Internet of Things (H-IoT) is an interconnection of devices capable of sensing and transmitting information that conveys the status of an individual’s health. The continuous monitoring of an individual’s health for disease diagnosis and early detection is an important application of H-IoT. Ambient assisted living (AAL) entails monitoring a patient’s health to ensure their well-being. However, ensuring a limit on transmission delays is an essential requirement of such monitoring systems. The uplink (UL) transmission during the orthogonal frequency division multiple access (OFDMA) in the wireless local area networks (WLANs) can incur a delay which may not be acceptable for delay-sensitive applications such as H-IoT due to their random nature. Therefore, we propose a UL OFDMA scheduler for the next Wireless Fidelity (Wi-Fi) standard, the IEEE 802.11be, that is compliant with the latency requirements for healthcare applications. The scheduler allocates the channel resources for UL transmission taking into consideration the traffic class or access category. The results demonstrate that the proposed scheduler can achieve the required latency for H-IoT applications. Additionally, the performance in terms of fairness and throughput is also superior to state-of-the-art schedulers.

## 1. Introduction

**Figure 1:**
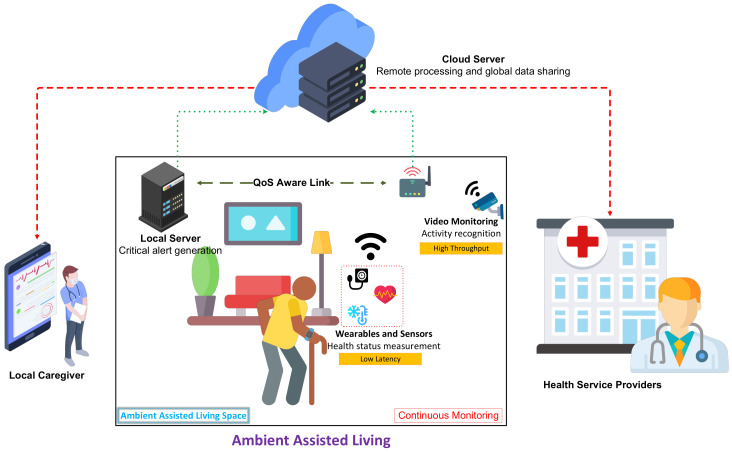
Continuous monitoring of patients in H-IoT: ambient assisted living.

The current demographic trends indicate that the world is moving towards an aging population. The World Health Organization (WHO) has predicted that one in six people will be over the age of 60 by 2030 [1]. The number of people aged over 60 will double by 2050. This aging trend has led to a growing research interest in ambient assisted living (AAL) systems. AAL is essentially assisted care for humans, especially at an advanced age or with severe medical conditions. The caregivers are assisted by technology, especially monitoring systems and socially assisted robots [2]. Internet of Things (IoT) is a key enabler in an AAL system. IoT can provide insights about the patient’s health and living environment and can ubiquitously monitor their well being [3,4]. An IoT-based AAL system consists of an array of sensors, which may be health sensors such as an electrocardiogram (ECG), glucometer, pulse oximeter or an activity sensor such as an accelerometer, inertial sensor or even a camera [5]. These sensors continuously sense and transmit their respective data to a processing unit which may be local or cloud to generate insights about the patient’s health. The end users, i.e., patients, caretakers and healthcare professionals can access these insights to advise the observed individuals and take a suitable course of action [6]. Integrating artificial intelligence (AI) and fog computing with these sensor networks can enable greater autonomy and accuracy in detection, especially in terms of activity recognition [7]. The smart homes equipped with sensors are also capable of high accuracy health monitoring, especially in an AAL scenario. Figure 1 depicts the scenario of an AAL environment. However, the effectiveness of this monitoring system is significantly affected by the communication performance between the sensors and the processing unit. The IEEE 802.15.6 is a standard defined for wireless body area networks (WBANs) [8], but Bluetooth, Zigbee, Wi-Fi and cellular networks are commonly used in the commercially available wearable devices such as smart watches and health trackers [9]. The IEEE 802.11 Wireless Local Area Networks (WLANs) or Wi-Fi networks are capable of delivering high throughput especially for health monitoring and smart home applications [10]. However, one of the key requirements of the healthcare applications is low latency, where the critical health data should be transmitted within a bounded time limit. Due to the random nature of the wireless links, the latency performance of Wi-Fi has not been suitable for healthcare applications. The Wi-Fi standard has evolved over time to deliver increased data rates but has been limited in its support for dense deployments and low latency applications. The introduction of multi-user techniques (MU), allowing multiple simultaneous transmissions from different stations, opened new ways to support a large number of connected devices by keeping a low collision probability and access delay. Orthogonal frequency multiple access (OFDMA) is one of the new MU mechanisms brought by the IEEE 802.11ax (Wi-Fi 6) amendment [11]. However, OFDMA, as defined in IEEE 802.11ax’s specifications, does not ensure the optimal performance in terms of delay, network throughput and scalability. Therefore, an efficient management of spectral resources i.e. transmission opportunities (TXOP) and assigned spectrum is required.

The upcoming IEEE 802.11be amendment is also known as the extremely high throughput (EHT) amendment. It will also be branded as Wi-Fi 7. There are a number of proposed incremental upgrades over Wi-Fi 6 and a number of new features under consideration. The IEEE P802.11-Task Group BE (TGbe) is tasked with defining EHT physical (PHY) and medium access control (MAC) layers for a major amendment in the next generation of WLANs. The Project Authorization Report (PAR) approved in 2019 defined the scope of this amendment to the IEEE 802.11 PHY and MAC layers [12]. The key modifications include:

- At least one operational mode that supports a minimum of 30 Gbps of maximum throughput at the service access point (SAP).
- Operation at the frequency range between 1 and 7.250 GHz.
- Backward compatibility with legacy standards operating at 2.4, 5 and 6 GHz frequency bands.
- At least one operational mode for worst-case latency and jitter.

However, the upcoming Wi-Fi 7 amendment is also set to have new features enabling time-sensitive networking (TSN) [13]. These proposed features will enable this amendment to support high quality-of-service (QoS) applications such as real-time health monitoring and remote robotic control [14]. OFDMA was introduced in the Wi-Fi 6 to improve the spectral efficiency and to support large deployments [15], but Wi-Fi 7 is expected to enhance the OFDMA performance by including new features in the OFDMA operation. Using an access point (AP) as a scheduler to allocate the spectral resources can affect the delay performance. The AP acts as a scheduler which can solicit uplink (UL) transmissions from the Wi-Fi stations (STAs) when it gains the TXOP. Therefore, an optimal delay performance can be achieved by utilizing an optimal scheduling methodology. Intel Corporation outlined potential approaches to enable TSN in WLANs [16], including defining a low-latency access category (LL-AC) that can provide a deterministic service with a defined latency. Additionally, a provisioning mechanism needs to be developed for the the LL-AC that would entail modifications in the current QoS-aware standards. Provisioning the resources using an AC-aware mechanism can bridge the gap between the performance of the current Wi-Fi standards and the performance requirements of delay-sensitive applications such as H-IoT. Some applications have well-defined bounds on their delay and reliability performance as outlined in [17]. Therefore, we propose a scheduling algorithm that utilizes a two-pronged approach, utilizing the buffer size of the different access categories (AC) and tracking the previous transmissions of the STAs. Since H-IoT data transmissions are sensitive to delay, we distinguish between the various traffic classes that are identified by their AC while scheduling UL transmissions from the STAs. This distinction allows the scheduler to determine which STAs have a higher priority over other STAs in transmitting their queued frames. This approach results in a lower latency for the higher priority traffic along with ensuring fairness among the STAs.

The literature review presented in Section 3 reveals that the QoS information remains under-utilized for scheduling transmissions using OFDMA in the Wi-Fi networks. Additionally, the new amendment to the existing Wi-Fi standards, IEEE 802.11be, is under development that considers TSN as a significant goal. Several research works list latency as a critical metric in healthcare-based IoT applications, and numerous works focus on improving the latency performance in context of healthcare-based applications [18,19,20,21]. The work [19] identifies scheduling transmissions in time as a potential strategy to reduce delay. Therefore, we can list the following as the contributions of this work:

- We propose an OFDMA scheduler that utilizes the priorities of various ACs for scheduling UL transmissions in delay-sensitive IEEE 802.11be-based IoT networks, especially for healthcare applications.
- We also evaluate the performance of the proposed methodology on a network simulator. NS-3, and compare its performance with state-of-the-art mechanisms.

The remaining part of this paper is structured as follows. Section 2 introduces the enhancements in the IEEE 802.11be amendment. Section 3 presents an overview of the related work. The proposed OFDMA scheduler is introduced in Section 4, and its performance evaluation is presented in Section 5. Finally, the authors conclude the discussion in Section 6. The term EHT is interchangeably used with the name of the amendment “IEEE 802.11be”. Table 1 lists the frequently used abbreviations used in the manuscript.

## 2. IEEE 802.11be Extremely High Throughput Standard

The IEEE 802.11be amendment will include modifications to the IEEE Std 802.11 PHY and MAC layer. These enhancements are aimed to increase the throughput to a minimum of 30 Gbps while considering the limits on latency for time-sensitive applications. The groundwork for these changes has already been laid in the IEEE 802.11ax standard, and this amendment builds upon them. Like its predecessor, EHT uses OFDMA for the allocation of bandwidth resources to the STAs associated with it. Authors in [22] offer a detailed discussion on the new and improved features of the EHT. These proposals can be divided into two sub-groups: enhancements at the PHY Layer and enhancements at the MAC Layer.

### 2.1. Enhancements at the PHY Layer

The role of the PHY layer in the WLANs is to define the electrical transmission specifications of the device and its interaction with the transmission medium. The PHY layer functions include modulation of the data, establishment of connection over a medium and allocation of resources among the participating nodes in the network. The proposed enhancements improve the transmission protocols and allocation of physical resources at the PHY layer. With the opening of the 6 GHz band, the available bandwidth has effectively doubled, and the TGBe is planning to exploit this newly opened spectrum. The 2.4 and 5 GHz uses a 20 MHz primary channel. In the 5 GHz band, up to 160 MHz channels are available in 80 + 80 MHz or 160 MHz configuration. Additionally, in the 6 GHz band, wider channel bands are available in multiple configurations. The 320 MHz channels can be realized by aggregating contiguous channels in the 6 GHz band entirely or by aggregating non-contiguous channels across 5 and 6 GHz bands [23]. The EHT is inclined to allow aggregations across the 2.4, 5 and 6 GHz bands. Therefore, EHT has included various schemes for channel aggregation configurations from across the different frequency bands such as contiguous 240 and 320 MHz bands, non-contiguous bandwidths in 160 + 160 MHz, or 240/160 + 80 MHz [24]. The tone plans for up to 160 MHz wide channels are expected to remain similar to the IEEE 802.11ax 5 GHz band. A 2018 document [25] of TGbe discusses the throughput enhancement utilizing multi-band transmissions.

The use of higher-order modulation schemes increases the throughput under favorable received signal quality. Therefore, as an evolutionary step, the recommendations for 4096-Quadrature Amplitude Modulation (QAM) is on the books [22]. The higher the order of the constellation is, the more information can be transmitted per symbol. The 4096-QAM allows 12-bit symbols that result in a 20% increase in the data rate compared to 1024-QAM in 802.11ax WLANs [26]. To achieve high SNR for 4K-QAM, STA will require multiple spatial streams (SS), therefore affecting the multi-user (MU) performance, which must sacrifice the benefits of spatial multiplexing in favor of spatial diversity. Therefore, this feature will require further exploration before being accepted by the EHT.

The PHY preamble design for the EHT will incorporate changes due to the added features, but at the same time, it should be able to maintain backward compatibility. Each preamble since the IEEE 802.11n standard can be identified by frame design, which would include modulation information. The EHT PHY frame will include a universal signal field (U-SIG) as well as EHT specific signal field (EHT-SIG). The U-SIG field is expected to introduce forward compatibility. However, some of the information will be version dependent, while some information will be version independent. The EHT-SIG field will convey information regarding the new features of the EHT standard [26].

### 2.2. Enhancements at the MAC Layer

Many significant MAC features from IEEE 802.11ax such as MU multiple input multiple output (MU-MIMO), OFDMA and spatial reuse will be extended in IEEE 802.11be. The support for more SS will also enable more flexible MU-MIMO arrangements. However, the current explicit channel state information (CSI) acquisition procedure may not cope well with such a high number of antennas, and for that reason, TGbe is currently evaluating several alternatives to enhance explicit sounding, even considering the introduction of an implicit procedure. As for OFDMA, enhanced resource unit (RU) allocation schemes will allow allocating multiple contiguous and non-contiguous RUs to a single STA. Consequently, these novel schemes could significantly increase spectral efficiency and overall network throughput and, even better, satisfy timely data delivery. Whether based on MU-MIMO or OFDMA, MU transmissions are key to reducing the channel access latency, as packets from/to different users can be de-queued simultaneously. MU transmissions reduce contention by minimizing the negative impact of collisions, which is exacerbated by the exponential backoff used in the legacy medium access. The multi-link operation (MLO) will likely become the most representative feature of IEEE 802.11be, being able to yield an order-of-magnitude reduction in the worst-case latency experienced by Wi-Fi devices and meet the stringent requirements of real-time applications (RTAs) even under dense traffic conditions [24,26,27].

## 3. Related Work

**Figure 2:**
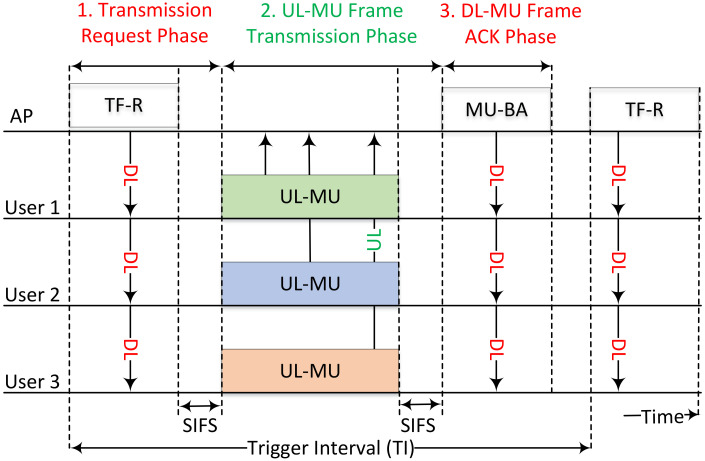
Trigger-based UL multi-user transmission sequence in IEEE 802.11ax WLAN.

**Figure 3:**
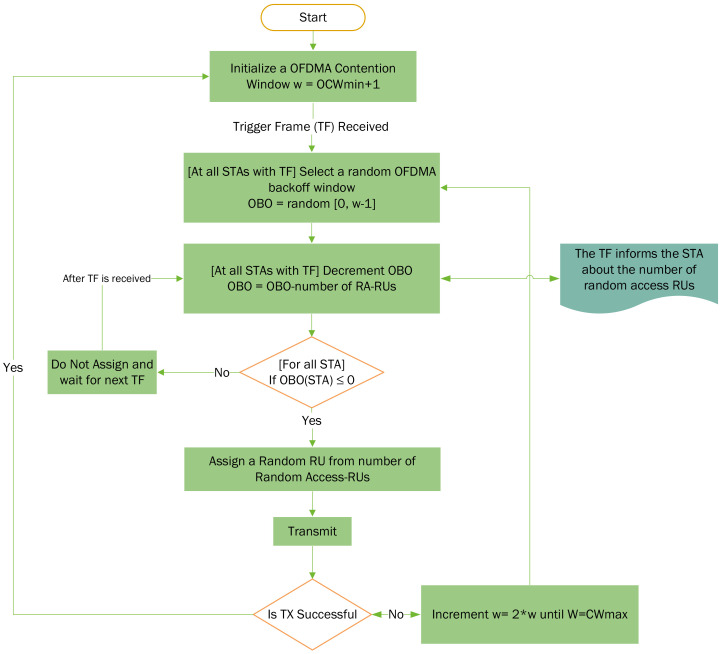
UORA operation for random access in UL-OFDMA.

The OFDMA essentially divides the resources, both in frequency and time, which enables the allocation of resources to multiple users at a time instant. In the frequency domain, a channel is divided into sub-carriers that are allocated for a time interval that is several symbols long. These sub-carriers are grouped in standard combinations. These combinations of sub-carriers or “tones” form the RUs which are allocated to multiple users. The IEEE 802.11ax was the first Wi-Fi standard to utilize the OFDMA. A 20 MHz channel in the 802.11ax is composed of 256 sub-carriers which are combined in groups of 26, 52, 106 and 242 tones. With the sub-carrier spacing of 78.125 kHz, these RUs constitute sub-channels of 2, 4, 8 and 20 MHz bandwidth, respectively. MU-OFDMA aims at improving the spectral efficiency of the wireless medium by allowing multiple STAs to transmit simultaneously during a TXOP by using different orthogonal frequency division multiplexing (OFDM) sub-carriers.The process of UL is depicted in Figure 2. The high-efficiency WLANs (HE-WLAN) use UL-OFDMA-based random access (UORA) for randomly selecting the STAs for UL transmission. UORA assigns the different RUs in UL. The RUs for random access (RA) are termed as random access resource units (RA-RUs) and have an have an Association ID (AID) value of 0. The RA-RUs for unassociated STAs have an AID equal to 2045. The STA in scheduled access (SA) do not contend for RUs in the RA process and are assigned a non-zero AID other than 0 or 2045. All STAs supporting UORA maintain an OFDMA backoff (OBO) counter that is randomly selected between 0 and OFDMA contention window (OCW) value. The OCW value, where $OCW\in (0,OCW_{max})$, is initialized at the $OCW_{min}$ value. The $OCW_{min}$ and $OCW_{max}$ values are conveyed by the AP using the UORA Parameter Set. The UL OFDMA process is initiated by the AP by sending a Trigger Frame (TF) that conveys the number of RA-RUs that are available for UL transmission [28]. Each STA decrements the number of RA-RUs from its respective OBO value. The STAs for which the difference $\left(OBO-N_{RA-RU}\right)$ is zero or a negative value are allowed to randomly select a RU from their respective set of eligible RA-RUs (AID 0 or 2045) to transmit the frame. The STAs with a non-zero difference retain this value for the next TF to attempt transmission using it as their OBO value. After a successful transmission, the OCW is set to the latest $OCW_{min}$ value from the latest available OFDMA Parameter Set. If the STA fails to successfully transmit a frame, it doubles its OCW each time until the $OCW_{max}$ is reached. The re-transmission may also be attempted by the simultaneous Enhanced Distributed Channel Access (EDCA). This process is depicted in Figure 3.

The authors in [11] evaluate the impact of OFDMA on the system throughput and latency in saturated and unsaturated conditions. The authors conclude, that in saturated conditions, throughput and latency performance is considerably degraded. However, the efficiency and throughput gains using OFDMA are maximized by introducing a scheduling mechanism at the AP. The work [29] analyzes the performance of various scheduling strategies. The UL-OFDMA scheduling is designed as an optimization problem, where a utility function associated with the RU allocation is maximized under constraints. The work in [29] also explores some classic schedulers to evaluate the effect on uplink data transmission rate. To enable real-time operation over a Wi-Fi network, UL OFDMA scheduling can be implemented. In enabling real-time operation, a sub-millisecond latency is highly desirable with a reliability of 99.999%. Due to the random nature of the backoff mechanism in UORA, the delay performance is highly unpredictable. Authors in [30] attempt to ensure real-time operation. The authors propose a Cyclic Resource Assignment (CRA) algorithm to minimize the delay for the RTA frames with high reliability. However, the authors assume that the collisions occur only if two STAs select the same RU during the random access (RA) period. If there are no collisions during the RA, *f* STAs with data frames are allocated RUs where *f* is the number of RA-RUs. However, if a collision occurs, the AP allocates the RUs cyclically to $\left(N_{RU}-f\right)$ STAs without contention in the next slot, where $N_{RU}$ is the total number of RUs. The cycle continues until all STAs are allocated a RU without collision. When the cycle ends, the UORA resumes its normal operation. However, the results also show that when the number of STAs becomes large, the delay performance worsens, especially in default UORA. The CRA algorithm for RTA STAs occupies more bandwidth resources than the non-RTA STAs, thus showing unfair behavior. In [31], the authors implement the MU-OFDMA on the NS-3 simulator to evaluate the throughput performance and to validate their analytical model. The authors assume that all the STAs in the basic service set (BSS) are HE-STAs and explicitly solicit a buffer status report (BSR) from the STAs. They consider two performance indicators: the network throughput and the BSR delivery rate and show that in IEEE 802.11ax networks there is a trade-off between these indicators, which can be regulated by changing the number of RUs allocated for UORA. The authors discuss the impact of the BSR delivery rate parameter on the throughput. The simulations and the analytical model closely resemble each other and reveal that the RA-RU allocation is detrimental to the throughput performance compared to the deterministic access. It is also noteworthy, that at the time of writing this paper, there is no validated NS-3 implementation of UORA.

The paper [32] is focused on optimizing the UORA OBO mechanism. In principle, the OBO is decremented by the number of RU that are available and conveyed in a TF. However, it is not an efficient approach considering the impact it has on dense deployments. The authors propose an Efficient-OBO mechanism for the UORA process that does not incur additional control overhead. Instead, the authors consider the probabilities of RUs being allocated and derive an idea about the network congestion. The OBO is decremented by a value determined by a congestion parameter that takes into consideration the network congestion during that time slot. This also ensures that the starvation of STAs is avoided. In [33], the authors propose a hybrid approach to increase the throughput by introducing a carrier-sensing approach as an additional backoff mechanism in the UL-OFDMA operation. The AP senses if all RUs are occupied and attempts to fill all the RUs in a TXOP. It determines if a RU is unallocated by using a p-persistent carrier sense multiple access (CSMA) trial over a RU. It ensures high throughput without considering the buffer status of the STAs or requiring any additional signaling, but it suffers from high latency.

The current literature points out the fact that the support for RTAs is a major goal in Wi-Fi 7 networks, and scheduling the UL transmissions can significantly improve the latency performance in a WLAN. It is evident from the literature that the QoS requirements of the STAs are not individually catered to, and neither is the BSR information widely exploited for scheduling the UL transmissions. Therefore, this work envisages devising delay-sensitive scheduling for UL OFDMA considering QoS traffic, while at the same time ensuring that the non-QoS STAs do not starve. The unexploited BSR information can allow the AP to schedule the transmissions based on the buffer status in the ongoing TXOP while considering their QoS requirements.

## 4. Delay-Sensitive OFDMA Scheduling for H-IoT in Wi-Fi 7

To ensure fulfillment of the delay requirements, an OFDMA scheduling algorithm is proposed for the H-IoT. The proposed algorithm takes the queue sizes of the various ACs into account in addition to the previous scheduling decisions to allocate an RU during UL OFDMA. Since the AP is aware of the queue size of the STAs, an AP can act as a scheduler in a BSS. In the proposed delay-sensitive scheduler, we assume that *n* STAs are associated with an AP. The STAs transmit and receive frames from the AP. There are two ways in which the STAs and AP can transmit data, either by gaining a TXOP using the EDCA or using OFDMA. The STAs and the AP all compete for TXOP using EDCA. However, when an AP gains the TXOP, it can communicate with the STAs using OFDMA, both in the UL and DL. This work focuses on the UL transmission from the STAs to the AP. The STAs transmit a BSR in response to a buffer status report poll (BSRP) transmitted by the AP. The BSR relays traffic information of each AC at the STAs to the AP, which can be utilized in making scheduling decisions. Additionally, the header of the QoS-Data frames transmitted by the STAs contains the queue size and the traffic identifier associated with it in the QoS Control Field. This work proposes utilizing this key information to schedule UL transmissions from STAs to minimize the transmission delay for time-sensitive data frames without utilizing additional signalling overhead.

**Figure 4:**
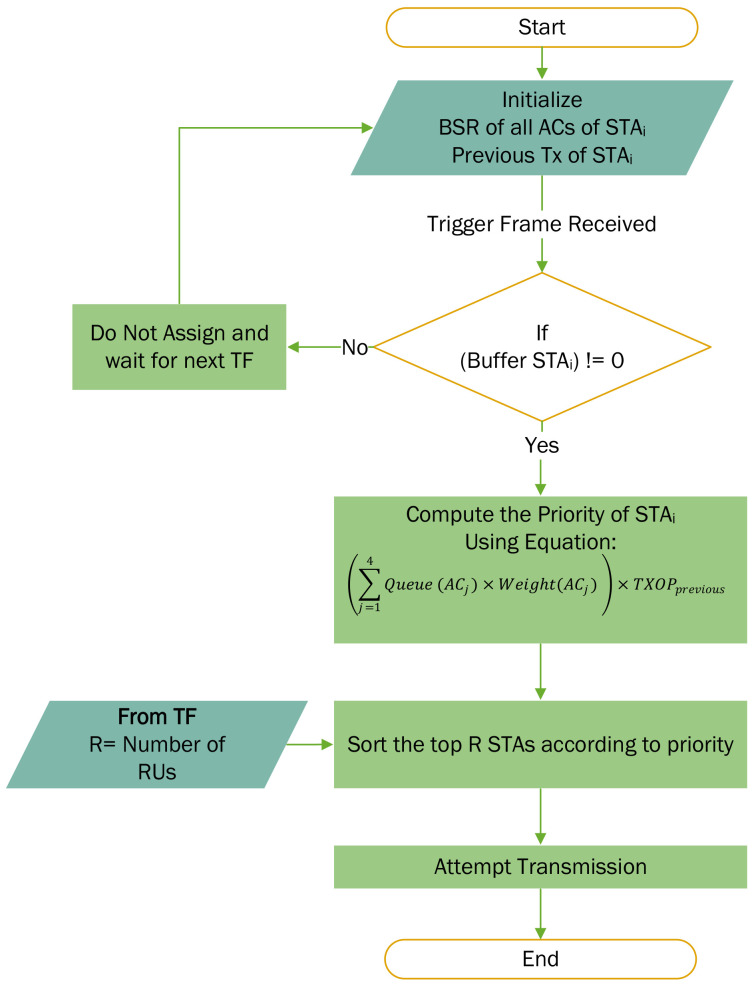
The flowchart of the proposed algorithm.

Each AC has a different priority that is enforced in the EDCA with the different EDCA parameter set values. However, in the case of OFDMA, there is no distinction between the different ACs. From the discussion in Section 3, it is clear that the scheduling for the UL transmission does not consider the queue sizes of different ACs. The proposed algorithm takes into confidence the following two parameters: (1) the queue size of each AC at the STA, and (2) previous transmissions at the STA. Initially, the scheduler initializes a priority against each STA that is based on the queue size of each AC and the previous transmissions. With each subsequent TXOP, the priority is updated to consider the current queue size of each AC. The higher priority ACs are given more consideration compared to the low priority ACs while calculating the priority. Additionally, the priority of the STA also considers the previous transmissions of an STA. The STAs with a non-zero queue size for higher priority AC and a history of transmission in previous TXOPs indicate that the STA has accumulated critical data frames. Equation (1) denotes the mathematical equation representing the priority calculation for each STA. Figure 4 illustrates the proposed algorithm as a flowchart.

(1) $$Priority\left(STA_{i}\right)=\sum_{j=1}^{4}Queue\left(AC_{j}\right)\times Weight\left(AC_{j}\right)\times TXOP_{Previous}\left(STA_{i}\right)$$

where $Queue(AC_{j})$ is the queue length of each AC, $Weight(AC_{j})$ is the weight or priority of each AC, and $TXOP_{Previous}\left(STA_{i}\right)$ is the indicator of previous transmissions by that STA. The list of ACs AC={AC_BE,AC_BK,AC_VI,AC_VO} includes the four ACs defined in the IEEE 802.11e standard [34]. These ACs correspond to the best effort, background, video and voice, respectively. Therefore, the STAs with the larger queue of high-priority AC traffic are given a higher priority. Additionally, the weight of each AC is determined by the use case which reflects the importance of each AC for that specific application. For the vital health monitoring applications in H-IoT, best effort traffic (AC_BE) has a greater significance over the voice traffic (AC_VO) or video traffic (AC_VI).

### Complexity Analysis

Since the time complexity of an algorithm is a function of the size of the input it takes, the time-complexity of the proposed scheduler depends on the number of STAs in the BSS. Equation (1) depicts the process of calculating the priority of an STA based on its traffic patterns and the previous transmissions. For *n* STAs in a BSS, the buffer status and previous transmissions are obtained and are then used to calculate the priority of each STA. The statement for calculating the priority has a complexity O(1) which is calculated *n* times for *n* STAs. Our implementation in NS3 uses an emplace function to arrange the STAs in an array according to the priority of the STA. The C++ emplace function has a complexity of O(n)[35]. Therefore, the overall time complexity of the algorithm is O(n), as the algorithm loops through *n* STAs without any nested operations.

## 5. Experimental Evaluation

To evaluate the performance of the proposed algorithm, an event-based network simulator for wireless networks, known as Network Simulator-3 (NS-3) is used. The NS-3 version 3.35 was utilized to simulate the proposed algorithm. Since the IEEE 802.11be is an incremental development over the 802.11ax standard, most of the basic functions remain common in both standards. Therefore, we evaluate the performance using 802.11ax implementation as a basis.

Section 3 discusses UORA, which is the default UL random access method in OFDMA. However, the UORA algorithm is not yet included in the NS-3. Therefore, we implement our version of UORA in NS-3 while considering some key assumptions. We assume that only a single RU can be allocated to a single STA in a TXOP and that the collision occurs when two STAs randomly select the same RU. However, we do not consider collision occurring during the transmission, and we limit the $OCW_{max}$ value range in between [32,1024]. We also compare our proposed method with the OFDMA scheduler presented in [11] and validated in NS-3. The scheduler in [11] uses a history-aware approach for scheduling the transmissions. The authors determine the priorities based on the previous allocations and then schedule transmissions in a round-robin fashion. The AP considers the previous transmissions between the AP and STA to determine a metric for scheduling the transmissions in DL and UL. Table 2 compares the focus of this work with the state of the art.

Equation (1) has three variables: queue size of each AC, the weight reflecting the priority associated with the corresponding AC and the previous transmissions of the STA. The weight of each AC is determined by the use case. Its value reflects the urgency of transmission of a frame belonging to an AC. In the evaluation, we consider two ACs: best effort (BE) and voice (VO), which are distinguished in their priority. The weight for each AC, denoted by $Weight(AC_{j})$ reflects which traffic class should be favoured over the other when making the scheduling decisions. We assign a value between zero and one to each AC while ensuring the sum of all the $Weight(AC_{j})$ values is equal to one. This implies that, if one AC has a higher priority, its queue size effects the STA’s priority more significantly than the AC with a lower $Weight(AC_{j})$ value. However, the $Weight(AC_{j})$ can be selected experimentally based on the traffic patterns. The offered load is generated as a UDP application of different AC by setting the traffic identifier (TID) value specific to a given AC. The transmissions are scheduled for both the DL and UL, but the UL transmission remains the focus of this work. Additionally, the RU size is dynamically selected for each TF but remains the same for all STAs during a TXOP. The simulator selects the number of RUs based on the number of STAs that have UL traffic during that TXOP, and the maximum number of available RUs is determined by the channel width. In this evaluation, the maximum number of RUs is 18 when 26-tone RUs are generated, as we utilize a 40 MHz channel. We evaluate the performance of the proposed algorithm according to three key metrics: latency, fairness and average throughput. We also evaluate the satisfaction levels for different applications in terms of delay performance. Additionally, we evaluate the delay performance as a function of the Modulation and Coding Scheme (MCS) index. Table 3 lays out the parameters that are used in the evaluation.

**Figure 5:**
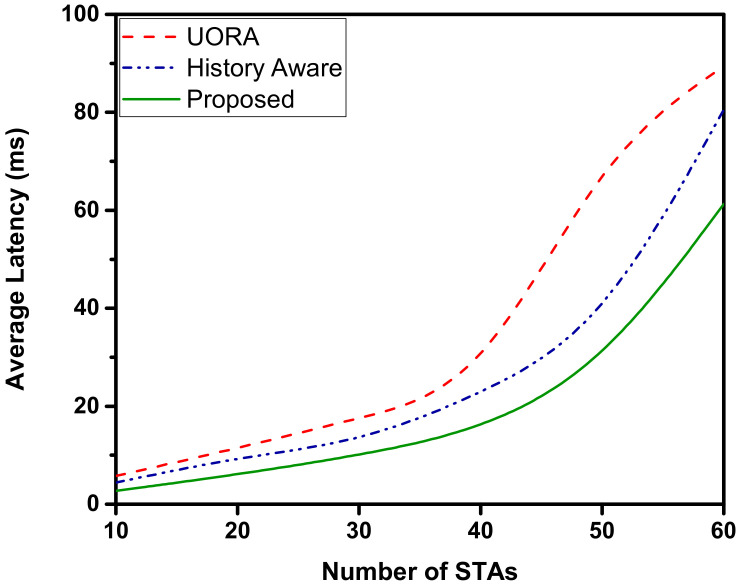
Average UL latency. As the number of STAs increases, the average latency increases.

Figure 5 represents the average latency in UL transmissions. For the proposed OFDMA scheduler, the latency up to 30 STAs remains below the 10 ms but increases with the further increase in the number of STAs. Comparatively, UORA has a significantly higher latency caused by the large number of STAs leading to an increased probability of collisions due to more STAs selecting the same RUs. The history-aware scheduler performs better than the UORA and is surpassed by our proposed OFDMA scheduler.

**Figure 6:**
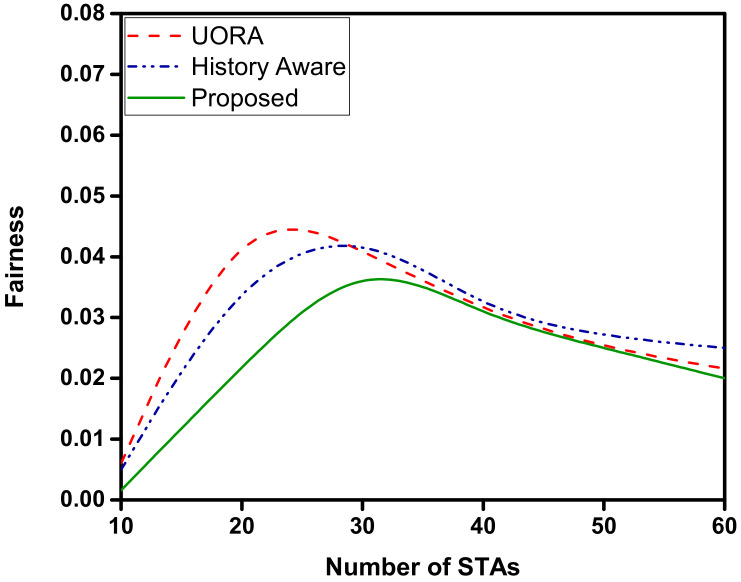
Fairness in RU allocation. The lower the value is, fairer the RU allocation will be.

**Figure 7:**
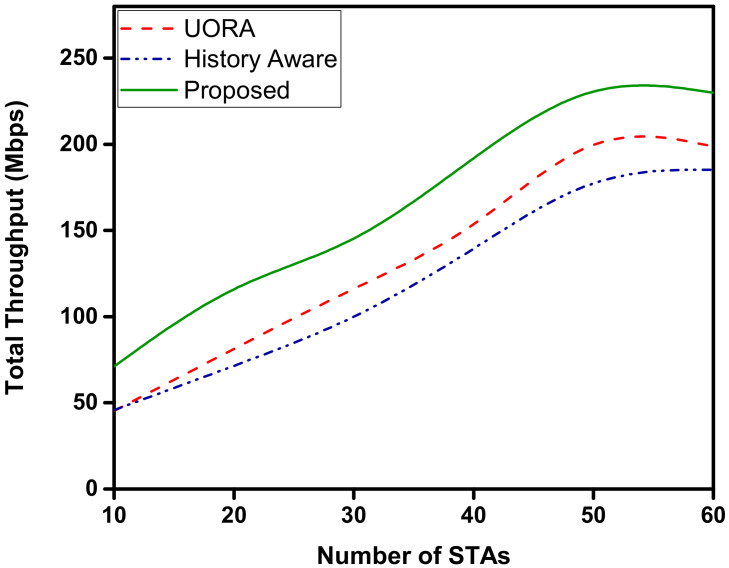
Average throughput achieved. The increase in the number of STAs increases the total average throughput.

Figure 6 illustrates the fairness of the allocation compared to the UORA and history aware scheduler. We compute the fairness as the standard deviation of the average throughput of each STA. Therefore, the lower the standard deviation is, fairer the algorithm will be. As shown in the figure, the performance of the proposed scheduler is significantly better than the other mechanisms. The allocation becomes fairer with the increasing number of devices as the distinction in priority becomes lower, owing to a larger number of devices having larger queues due to scheduling delays. However, at the lower number of STA, the scheduling delay is lower; therefore the difference in priorities between the STAs is comparatively significant. This leads to a relative unfair allocation of RUs at lower number of STAs. Therefore, for STAs with similar traffic patterns, the proposed scheduler acts with fairness. In the case of UORA, the allocations are random; therefore the allocation may be lopsided and cause unfairness in the short term. For the history-aware mechanism, the allocations are made based on the previous transmissions, which may cause some of the STAs to be starved when they are not allocated an RU in the initial TXOPs. Moreover it is compounded with every TXOP, as the priority is calculated using channel resource allocation in time units. The throughput performance is shown in Figure 7. The throughput of the proposed OFDMA scheduler is higher than UORA and the history-aware scheduler. The proposed OFDMA scheduler can maintain a higher average throughput owing to its knowledge of the queue sizes of the STAs. The throughput tends to saturate when the number of STAs is increased beyond 40 STAs. However, it fares better compared to the other two mechanisms.

**Figure 8:**
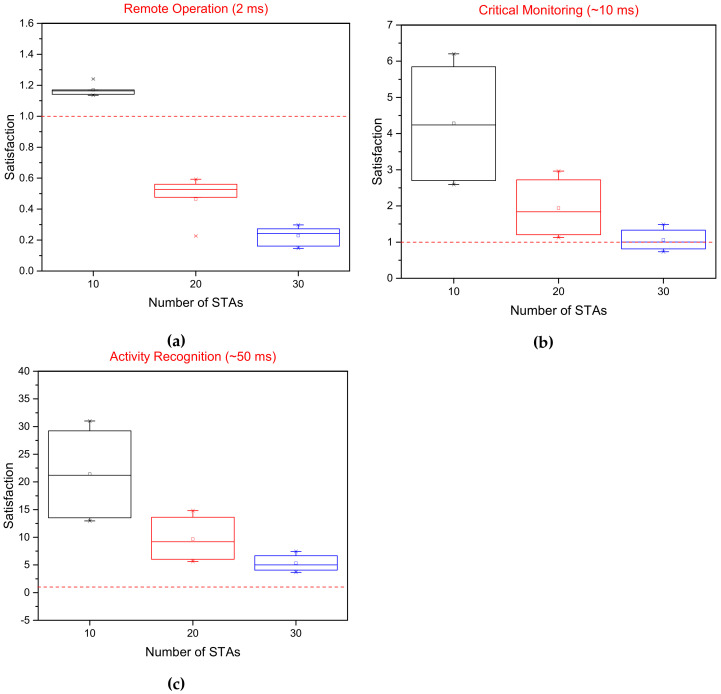
Satisfaction level of the proposed OFDMA scheduler in terms of delay for three different application classes in H-IoT. The red line at 1 depicts the threshold value for meeting the satisfaction levels of different applications. (**a**) Remote control applications with haptic control. (**b**) Critical monitoring of health parameters in real-time. (**c**) Activity recognition using real-time video.

**Figure 9:**
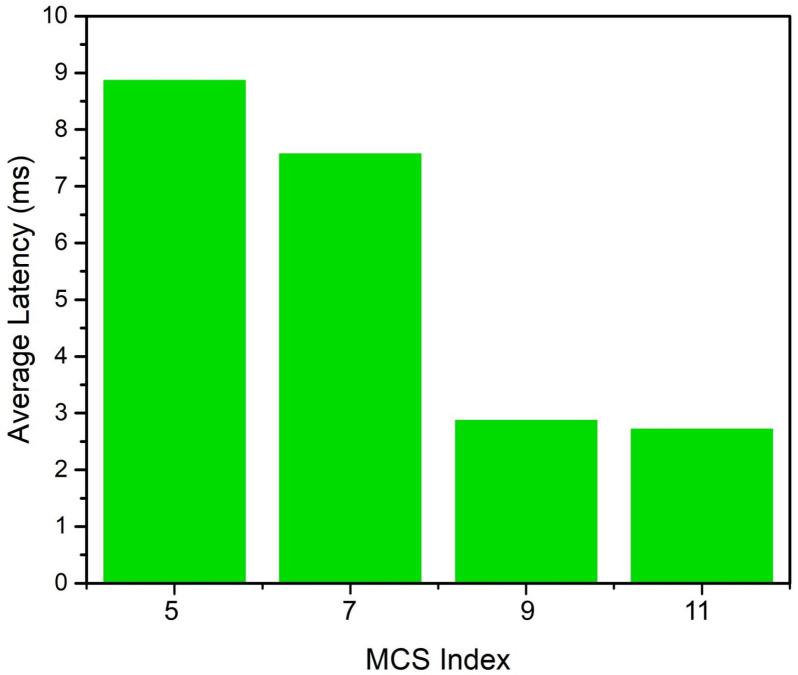
Impact of MCS index on the average latency.

The evaluation and its analysis shows that scheduling the transmissions while considering the traffic categories can significantly improve the performance of a QoS-aware network. The improvements, in terms of latency, can enable new applications, especially in the healthcare sector where timely monitoring can be of critical importance. Compliance with the QoS requirements can be ensured by exploiting the queue size information without incurring any additional overhead. Different H-IoT applications impose different QoS requirements in terms of delay as stated in [18]. Those requirements are used in our evaluation to establish a level of satisfaction for the each application. Figure 8a–c represent the satisfaction level in terms of delay for different H-IoT applications. The proportion of traffic flows above the line at satisfaction value one satisfies the requirements of a given application. Figure 8a shows the proportion of traffic flows that satisfies the requirements for remote control applications such as haptic control for remote robotic operation using the proposed OFDMA scheduler. Figure 8b,c depict the performance of the proposed scheduler for remote monitoring applications. The performance of patient monitoring applications and real-time health and activity monitoring is comfortably within the acceptable range. With the increase in the number of STAs, the delay increases due to a limited number of RUs which can be allocated. The MCS index determines the performance of a Wi-Fi network while considering numerous factors such as the data rate, number of SS, modulation scheme, error correction and channel width. Therefore, the impact of the MCS index on latency performance is presented in Figure 9. It is observed that with the increase in the MCS index value in the given 40 MHz channel and constant guard interval, the latency performance improves. Higher MCS values correspond to higher order modulation and error coding schemes.

## 6. Conclusions

The upcoming IEEE 802.11be standard is the next iteration in the IEEE 802.11 family of WLAN standards. The introduction of OFDMA in the IEEE 802.11ax standard enabled large deployments, thereby enabling more applications, especially for IoT. To support a wider range of applications, additional enhancements are being proposed to the currently developing amendment. Scheduling transmissions between the AP and STAs is a critical operation that significantly affects the performance of the WLANs, especially for UL transmissions in QoS-bound applications. The traffic in a WLAN is classified into various ACs which have different priorities, and these priorities are enforced in EDCA but not in OFDMA. The AP can act as a scheduler to allocate RUs for transmissions using their priorities as an allocation criteria. Therefore, we propose scheduling the UL transmissions based on the priority determined by the queue size of each AC and the weight associated with each AC. We evaluate the performance of the proposed OFDMA scheduler using the NS-3 simulator. We observe that the performance, in terms of latency, fairness and throughput is superior compared to the state of the art. The proposed scheduler can enable H-IoT applications, such as continuous health monitoring for real-time disease diagnosis and AAL. We plan on extending this work by including artificial intelligence in the scheduling mechanism for the upcoming IEEE 802.11be standard.

## Figures and Tables

**Table 1:** List of abbreviations used in the manuscript.

Abbreviation	Description
AAL	Ambient Assisted Living
AC	Access Category
AID	Association ID
AP	Access Point
BSR	Buffer Status Report
BSRP	Buffer Status Report Poll
BSS	Basic Service Set
CW	Contention Window
EDCA	Enhanced Distributed Channel Access
EHT	Extremely High Throughput
IoT	Internet of Things
MCS	Modulation and Coding Scheme
OCW	OFDMA Contention Window
OFDMA	Orthogonal Frequency Division Multiple Access
QAM	Quadrature Amplitude Modulation
QoS	Quality-of-Service
RTA	Real-time Applications
RU	Resource Units
STA	Wi-Fi Station
TSN	Time-Sensitive Networking
TXOP	Transmission Opportunity
WLAN	Wireless Local Area Network

**Table 2:** Comparison of the proposed algorithm with the state of the art.

Scheduler	Latency	Throughput	Fairness
Proposed	✓	✓	✓
History Aware		✓	
UORA		✓	

**Table 3:** Parameters used in the simulation.

Parameter	Value
Frequency Band	5 GHz
Channel Width	40 MHz
Number of RUs	1, 2, 4, 8, 18
MCS Value	11
Guard Interval	0.8 μs
Number of STAs	10, 20, 30, 40, 50, 60
UORA CW range	[32, 1024]
Packet Size	1472 bytes

## Data Availability

Not applicable.
